# A costing analysis of B-GAP: index-linked HIV testing for children and adolescents in Zimbabwe

**DOI:** 10.1186/s12913-021-07070-3

**Published:** 2021-10-12

**Authors:** Arthi Vasantharoopan, Hendramoorthy Maheswaran, Victoria Simms, Chido Dziva Chikwari, Tariro Chigwenah, Rudo Chikodzore, Khulamuzi Nyathi, Gertrude Ncube, Rashida A. Ferrand, Lorna Guinness

**Affiliations:** 1 Department of Infectious Disease Epidemiology, London School of Hygiene and Tropical Medicine, London, UK; 2 Institute for Global Health Innovation, Imperial College London, London, UK; 3 MRC International Statistics and Epidemiology Group, London School of Hygiene and Tropical Medicine, London, UK; 4 Biomedical Research and Training Institute, Harare, Zimbabwe; 5 Department of Clinical Research, London School of Hygiene and Tropical Medicine, London, UK; 6 Health Economics Unit, University of Cape Town, Cape Town, South Africa; 7 Matebeleland South, Ministry of Health and Child Care, Bulawayo, Zimbabwe; 8 City Health Department, Bulawayo City Council, Bulawayo, Zimbabwe; 9 Ministry of Health and Child Care, Harare, Zimbabwe; 10 London School of Hygiene and Tropical Medicine, London, UK

**Keywords:** HIV, Index-linked HIV testing, Community-based HIV testing, Home-based HIV testing, HIV assisted-testing, Costing analysis

## Abstract

**Background:**

By testing children and adolescents of HIV positive caretakers, index-linked HIV testing, a targeted HIV testing strategy, has the ability to identify high risk children and adolescents earlier and more efficiently, compared to blanket testing. We evaluated the incremental cost of integrating index-linked HIV testing via three modalities into HIV services in Zimbabwe.

**Methods:**

A mixture of bottom-up and top-down costing was employed to estimate the provider cost per test and per HIV diagnosis for 2–18 year olds, through standard of care testing, and the incremental cost of index-linked HIV testing via three modalities: facility-based testing, home-based testing by a healthcare worker, and testing at home by the caregiver using an oral mucosal transudate test. In addition to interviews, direct observation and study process data, facility registries were abstracted to extract outcome data and resource use. Costs were converted to 2019 constant US$.

**Results:**

The average cost per standard of care test in urban facilities was US$5.91 and US$7.15 at the rural facility. Incremental cost of an index-linked HIV test was driven by the uptake and number of participants tested. The lowest cost approach in the urban setting was home-based testing (US$6.69) and facility-based testing at the rural clinic (US$5.36). Testing by caregivers was almost always the most expensive option (rural US$62.49, urban US$17.49).

**Conclusions:**

This is the first costing analysis of index-linked HIV testing strategies. Unit costs varied across sites and with uptake. When scaling up, alternative testing solutions that increase efficiency such as index-linked HIV testing of the entire household, as opposed to solely targeting children/adolescents, need to be explored.

**Supplementary Information:**

The online version contains supplementary material available at 10.1186/s12913-021-07070-3.

## Background

Since 2010, 1.4 million new HIV infections in children have been averted worldwide, while HIV mortality among children has halved [1, 2]. Despite this progress, 150,000 children were newly infected in 2019, falling short of the 2018 target to reduce new HIV infections in children to 40,000 [3]. In addition, HIV treatment coverage among children is significantly lower than among adults, likely due to higher levels of under-diagnosis [4]. Furthermore, many children present to clinical services and start antiretroviral therapy (ART) in older childhood and adolescence when they have developed advanced disease, with consequent poorer outcomes [5]. Notably, adolescents are the only age-group in whom HIV-related mortality has not declined [5–8]. HIV testing and counselling approaches aimed at the timely diagnosis and linkage to care of children and adolescents in high HIV prevalence settings are therefore urgently needed.

Index-linked testing refers to screening family, household or other contacts of a case for a disease. Index-linked HIV testing has been widely used to identify higher risk individuals, and thus a high-yielding strategy for HIV testing [9]. In sub-Saharan Africa children and adolescents living in households with known HIV-positive adults are more likely to be HIV-positive, and are often untested and untreated [9–11]. Index-linked HIV testing (ILHIVT), whereby children living with adults with HIV are targeted for testing, has the potential to identify high-risk, difficult to access children and adolescents, and to improve yield [9, 12, 13].

While WHO guidelines recommend offering testing to children of HIV-positive adults, countries in Eastern and Southern Africa have yet to integrate this policy into routine service delivery [9, 12, 14]. The paucity of data on effectiveness, cost and cost-effectiveness of ILHIVT may be one factor preventing its scale up. As there are no costing studies of ILHIVT within sub-Saharan Africa, the cost implications and cost-effectiveness of including this strategy within HIV service delivery programs is unknown.

The Bridging the Gap in HIV testing and care for Children in Zimbabwe (B-GAP) study assessed the effectiveness and cost of a multi-option ILHIVT strategy in both urban and rural settings in Zimbabwe [14]. This paper is the cost analysis of B-GAP estimating the cost of providing standard of care (SoC) HIV testing –voluntary, facility based HIV testing and counselling (HTC) – comparing to the incremental cost of different ILHIVT strategies.

## Methods

### B-GAP HIV testing intervention

In the B-GAP study, individuals with HIV enrolled in and attending care at study clinics, i.e. indexes, were offered HIV testing for any children and adolescents (aged 2–18 years) of unknown HIV status in their household by study staff. Indexes could choose one of three testing options for the child (ren)/adolescent(s): Clinic-based diagnostic testing using a rapid test; home-based rapid diagnostic testing by a healthcare provider; or home-based, caregiver-provider testing using an oral mucosal transudate (OMT) test. All participants diagnosed with HIV were linked to care at their nearest healthcare facility.

### Study setting

ILHIVT was provided at 9 of the 36 primary health care facilities in Bulawayo and Mangwe district in Matebeleland South province in Zimbabwe, (6 urban, 3 rural) selected based on size and accessibility [14]. Adult HIV prevalence in both these provinces is approximately 20% [14]. The facilities do not routinely employ ILHIVT. Cost data collection took place at 2 urban facilities in Bulawayo, and 1 rural facility located in the Mangwe, sampled by convenience (Table 1).

**Table 1:** Characteristics of costing study facilities, as of Sept 2018

	Clinic A	Clinic B	Clinic C
District	Bulawayo	Bulawayo	Mangwe
Setting	Urban	Urban	Rural
Catchment Area Population Total	42,497	31,492	9137
Catchment Area Population Under 15 yrs	14,433	10,696	4066
Catchment Area Population 15 years +	28,063	20,796	5071
Overall Facility Visits in 1 Year^a^	50,778	77,558	5118
HIV Tests Conducted in 1 Year^a^	2541	3276	1860
Number of people on ART as of Sept 2018	4478	4625^b^	960

^a^Based on one year of facility registries: Oct 2017 – Sept 2018

^b^Due to missing records, this tally is current as of June 2017

### Costing methods

We estimated the provider cost of facility level HIV testing and the provision of index-linked testing, following the Global Health Cost Consortium costing guidelines [15]. A combination of bottom-up and top-down costing was employed. First, we estimated the full cost of testing and diagnosis of children and adolescents through SoC facility testing for a 4-month time period before the B-GAP intervention (May– August 2018). We then estimated the incremental cost of ILHIVT and diagnosis of participants (September– December 2018) at the same 3 clinics, provided by the three modalities: clinic, health-care worker testing in the household, and caregiver-testing in the household. Costs estimated included personnel, consumables, overheads, building, equipment, training, start-up and transaction costs, but excluded any research related costs.

To gauge personnel time, we undertook a combination of direct observation and face-to-face interviews with clinic and B-GAP staff to quantify resources utilized to deliver all HIV testing models. We used prospective time-tracking diaries and direct observation to measure human resource time spent on different activities. Appendix 1 provides a summarized breakdown of time spent on ILHIVT activities. Prices of test kits were taken from the National Pharmaceutical Corporation of Zimbabwe (NatPharm), as specified by the centralized Matebeleland South pharmacy system. Overheads and other shared clinic costs were allocated to the testing activities based on area of the clinic utilized by each department and patient load depending on the line item.

All resources used were converted into costs using financial data collected from B-GAP project accounts, Bulawayo City Council and the district and provincial medical offices in Matebeleland South. Salaries of study staff who conducted ILHIVT, were substituted with those of facility primary care counsellors who would carry out this work in routine clinical settings. Equipment, building, training and start-up costs were annualized using expected length of life determined by WHO cost effectiveness and strategic planning prices for tradable goods, and discounted at a rate of 3% [16]. Due to the instability of the Zimbabwean currency, we estimated costs in US dollars. We converted all real-time gross settlement (RTGS) dollars, the Zimbabwean currency, into USD using the exchange rate at the time when the financial data was provided, using the Reserve Bank of Zimbabwe exchange rates [17]. All costs were converted to 2019 constant USD using the GDP deflator for the United States [18]. A detailed description of the cost data collection methods is provided in Appendix 2 and 3.

### Healthcare outputs

We used clinic registers to determine the outputs of the standard facility-based HIV testing service including numbers of: tests administered; HIV positive test results; tests administered to 2–18 year olds; positive test results among 2–18 year olds. We used B-GAP study data to determine the outputs of the three ILHIVT modalities, including the number of: index cases screened; children and adolescents identified through index cases; children and adolescents tested by the three modalities; HIV-positive children and adolescents diagnosed.

### Data analysis

Unit cost per SoC HIV test in clinics, and per diagnosis at each clinic were calculated by dividing the total 4 month costs of testing at each clinic by the total number of people tested, and the number identified as positive, respectively. As no additional resources were consumed at clinics to test children/adolescents we assumed the cost per test to be the same as for adults.

The incremental cost of ILHIVT was calculated by assessing the additional personnel time and resources to follow-up cases and test them. To obtain the incremental cost per test, the incremental cost was divided by the total number of children and adolescents who were tested using the respective modality. The cost per diagnosis of a positive child/adolescent was calculated as the incremental cost per test divided by the number who tested positive. The relationship between uptake and unit cost was explored by plotting the incremental ILHIVT cost per test, against uptake per modality.

### Sensitivity analysis

A univariate sensitivity analysis was performed on input variables for which there was a degree of uncertainty, or that constituted a significant portion of the total costs including the exchange rate and transaction costs. The impact of the RTGS conversion rate used (1USD:4 RTGS) was assessed by varying the conversion rate to the highest (1USD:1 RTGS) and lowest (1USD: 50 RTGS) observed rates during the study period. The impact of assumptions made around resource use items such as staff salaries (±5–10%,), building (±10–20%), equipment (±10–20%) and overhead costs (±10–20%) and the frequency of the refresher training (1/year – 4/year) were also tested. In addition, HIV prevalence of clinic attendees was varied from 5 to 20%, while yield of ILHIVT was varied from 2 to 7%. Finally, transaction costs defined as management costs incurred through non-governmental organization (NGO) supervision of intervention activities (for study staff), was varied (±10–20%).

### Scenario analysis

HIV self-testing is recommended by the WHO as an alternative HIV testing strategy for scale-up [19–21], and qualitative work conducted after the conclusion of the ILHIVT intervention indicated that lack of exposure to, and acceptability of caregiver-provided testing affected the uptake of this modality across all sites. We explored variations in the uptake of index-linked caregiver provided HIV testing to reflect potential real-world acceptability and implementation scenarios: 1.) equal uptake of all 3 modalities; 2.) a 50–50 split between facility based testing and testing performed by caregivers; 3.) 100% uptake of caregiver-provided testing at all clinics. Additionally, we compared the efficiency of home-based and caregiver-provided testing (i.e. non facility-based options) by identifying the uptake of caregiver provided tests required to match the unit cost of home-based ILHIVT performed by health care workers.

## Results

### Cost composition

Table 2 presents recurrent and capital costs for SoC HIV testing and the three ILHIVT modalities. The total monthly cost of providing SoC testing ranged from US$997 to US$1410. Across these facilities, personnel costs accounted for 56.9 to 70.9% of total costs; testing-specific consumables accounted for 27.9 to 35.7%; overheads from 1.2 to 9.8%, and less than 1% of total costs were attributable to capital resource items. The total incremental monthly cost of providing ILHIVT ranged from $379 to $400 in clinic; $359 to $420 for home-based testing; $255 to $288 for caregiver-provided testing. Across all three clinics and modalities; personnel costs accounted for 39.7 to 62.4% of total costs; testing-specific consumables accounted for 4.5 to 23.1%; transaction costs from 17.6 to 29.1%; capital resource items for 2.6 to 6.4%.

**Table 2:** Monthly cost breakdown of providing: 1.) Full SoC HTS at 2 urban and 1 rural clinic in Bulawayo and Mangwe District in Matebeleland South Province, Zimbabwe; 2.) Incremental ILHIVT according to 3 modalities – clinic, home-based and caregiver – at the same 3 clinics

	Clinic A – Bulawayo (Urban)				Clinic B – Bulawayo (Urban)				Clinic C – Mangwe (Rural)			
	SoC	Clinic	Home-Based	Caregiver	SoC	Clinic	Home-Based	Caregiver	SoC	Clinic	Home-Based	Caregiver
**Recurrent**	**$1178.43(99.4%)**	**$375.71 (93.9%)**	**$385.34 (96.6%)**	**$265.01 (97.2%)**	**$1401.98 (99.4%)**	**$355.07 (93.6%)**	**$346.30 (96.6%)**	**$280.52 (97.4%)**	**$993.20 (99.6%)**	**$357.15 (93.7%)**	**$407.82 (97.1%)**	**$246.97 (97.0%)**
Personnel	$706.06 (59.5%)	$219.60 (54.9%)	$221.32 (55.7%)	$108.08 (39.7%)	$802.19 (56.9%)	$236.76 (62.4%)	$187.00 (52.2%)	$125.24 (43.5%)	$704.39 (70.9%)	$167.96 (44.0%)	$253.02 (60.2%)	$128.01 (50.3%)
Consumables – (Test specific)	$352.56 (29.7%)	$54.90 (13.7%)	$63.23 (15.9%)	$56.14 (20.6%)	$503.63 (35.7%)	$17.10 (4.5%)	$58.50 (16.3%)	$54.48 (18.9%)	$276.77 (27.9%)	$87.98 (23.1%)	$54.00 (12.9%)	$18.16 (7.1%)
Consumables – (Other)	$3.17 (0.3%)	$27.14 (6.8%)	$26.72 (6.7%)	$26.72 (9.8%)	$2.38 (0.2%)	$27.14 (7.2%)	$26.72 (7.5%)	$26.72 (9.3%)	$0.05 (0.0%)	$27.14 (7.1%)	$26.72 (6.4%)	$26.72 (10.5%)
Overhead	$116.64 (9.8%)	NA	NA	NA	$93.77 (6.7%)	NA	NA	NA	$11.99 (1.2%)	NA	NA	NA
Transaction Costs (NGO Management)	NA	$74.07 (18.5%)	$74.07 (18.6%)	$74.07 (27.2%)	NA	$74.07 (19.5%)	$74.07 (20.6%)	$74.07 (25.7%)	NA	$74.07 (17.6%)	$74.07 (17.6%)	$74.07 (29.1%)
**Capital**	**$7.55 (0.6%)**	**$24.24 (6.1%)**	**$12.27 (3.1%)**	**$7.59 (2.8%)**	**$7.89 (0.6%)**	**$24.24 (6.4%)**	**$12.71 (3.4%)**	**$7.59 (2.6%)**	**$4.14** **(0.4%)**	**$24.24 (6.4%)**	**$12.27 (2.9%)**	**$7.59 (3.0%)**
Equipment	$4.33 (0.4%)	$13.50 (3.4%)	$1.53 (0.4%)	$0.00 (0.0%)	$2.51 (0.2%)	$13.50 (3.6%)	$1.53 (0.4%)	$0.00 (0.0%)	$1.71 (0.2%)	$13.50 (3.5%)	$1.53 (0.4%)	$0.00 (0.0%)
Building	$3.22 (0.3%)	NA	NA	NA	$5.37 (0.4%)	NA	NA	NA	$2.44 (0.2%)	NA	NA	NA
Intervention Start-Up	NA	$10.74 (2.7%)	$10.74 (2.7%)	$7.59 (2.8%)	NA	$10.74 (2.8%)	$10.74 (3.0%)	$7.59 (2.6%)	NA	$10.74 (2.8%)	$10.74 (2.6%)	$7.59 (3.0%)
**Total**	**$1185.98**	**$399.95**	**$397.60**	**$272.60**	**$1409.86**	**$379.30**	**$358.56**	**$288.11**	**$997.34**	**$381.39**	**$420.08**	**$254.56**

### HIV testing outcomes

From May 2018 to August 2018, 2317 clinic based SoC HIV tests were administered (Table 3). The test yield (proportion of test results that were positive), was 12.9% (299/2317 total tested). Across the 3 clinics 11.5% (267/2317) of those tested were 2–18 years of age, and the yield was 3.7% (10/267).

**Table 3:** Unit cost of the various HIV testing modalities across all three costing study clinics, over a 4 month time-period

Testing Modality	No. Tested	No. Positive	Cost Per Test (USD)	Cost Per Diagnosis (USD)
Clinic A – Bulawayo (Urban)				
Standard of Care (SoC) – Total	804	77	$5.90	$61.61
SoC 2–18 years	113	4	$5.90	$166.69
Index-Linked-Clinic	212	2	$7.41	$785.50
Index-Linked-Home-Based	231	1	$6.69	$1545.41
Index-Linked-Caregiver	62	0	$17.59	N/A
Clinic B – Bulawayo (Urban)				
Standard of Care – Total	955	157	$5.91	$35.92
SoC 2–18 years	71	3	$5.91	$139.76
Index-Linked-Clinic	48	1	$31.08	$1492.02
Index-Linked-Home-Based	191	0	$7.18	N/A
Index-Linked-Caregiver	51	0	$21.63	N/A
Clinic C – Mangwe (Rural)				
Standard of Care – Total	558	65	$7.15	$61.37
SoC 2–18 years	83	3	$7.15	$197.80
Index-Linked-Clinic	263	4	$5.36	$352.59
Index-Linked-Home-Based	189	2	$8.65	$817.21
Index-Linked-Caregiver	16	1	$62.40	$998.41

1. Unit cost of SoC HIV testing presented for all clinics span May-Aug 2018 period

2. Incremental unit cost of index-linked testing for all modalities, presented for all clinics span Sep-Dec 2018

The 3 clinics screened 2087 index-cases, identified 1708 eligible children and adolescents of unknown HIV status, and of those, tested 1263 (74%); 41% in clinic, 48% via home-based testing, and 10% through assisted testing. Uptake of each modality varied by clinic; home-based testing was the preferred option at urban clinics A (45.7%) and B (65.9%), while clinic testing was the preferred option at rural clinic C (56.2%). The yield of ILHIVT at Clinics A, B and C was 0.6% (3/505), 0.3% (1/290) and 1.5% (7/468) respectively. Yield by modality is as follows: 1.3% (7/523) via clinic; 0.5% (3/611) via home-based; 0.8% (1/129) via caregiver-assisted testing (Table 3).

### Unit costs

The cost per child/adolescent tested through SoC ranged from US$5.90 to US$7.15. The cost per positive SoC test result ranged from US$ 35.92 to US$61.61. The costs per HIV-positive child or adolescent, 2–18 years of age identified through SoC were US$166.69, US$139.76, US$197.80 at Clinic A, B and C respectively (Table 3).

**Fig. 1:**
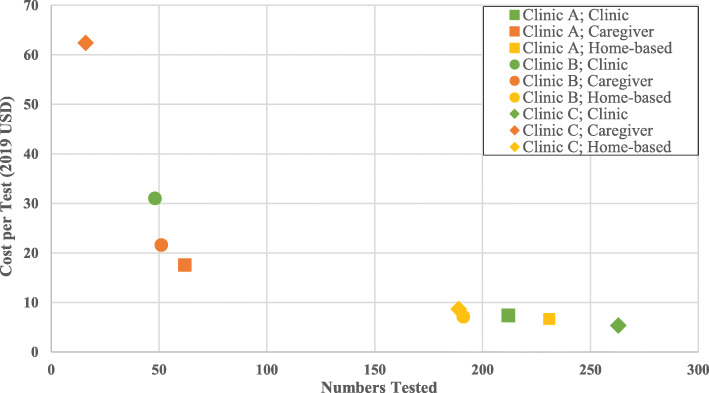
Uptake of *Index-Linked HIV Testing* vs associated *Incremental Cost per Test*, according to modality: Clinic; Caregiver; Home-Based

The average incremental cost per child/adolescent tested through ILHIVT at the clinic ranged from US$10.56 (urban clinic A) to US$25.47 (rural clinic C). The incremental cost per modality across all clinics ranged as follows: clinic – US$5.36 (clinic C) to US$31.08 (clinic B); home-based testing by health care worker – US$6.69 (clinic A) to US$8.65 (clinic C); caregiver-provided testing – US$17.59 (clinic A) to US$62.40 (clinic C). The cost per diagnosis of an HIV positive child/adolescent through ILHIVT ranged from US$352.59 to US$1492.02 for clinic based ILHIVT and US$817.21 to US$1545.41 via home-based testing by health care worker. Clinic C had the only positive test result from caregiver-provided testing, which cost US$998.41 (Table 3). The uptake of ILHIVT via modality and associated incremental cost per test indicates that costs are likely dependent on quantity: the fewer tests administered via modality, the higher the associated incremental unit cost per test, and vice versa (Fig. 1).

### Sensitivity analysis

**Fig. 2:**
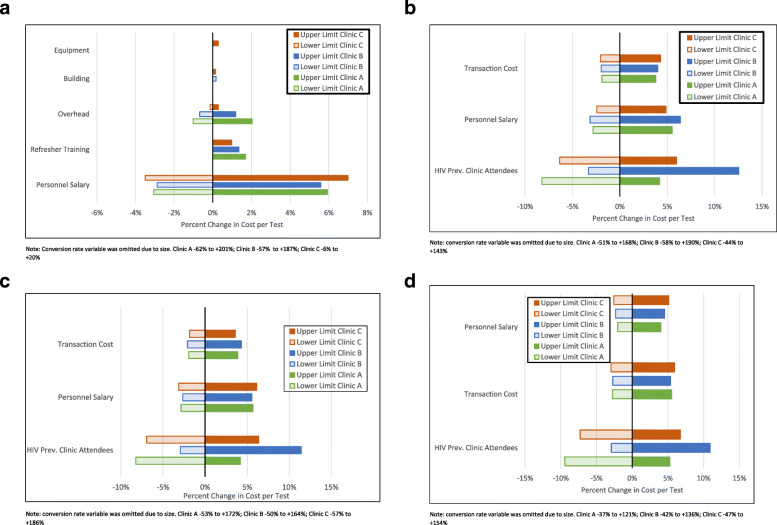
**a.** Tornado plot of model parameters varied in univariate sensitivity analysis of Adolescent SoC HTS and impact on Cost per Test. **b.** Tornado plot of model parameters varied in univariate sensitivity analysis of Index-Linked Testing via Clinic modality and impact on Cost per Test. **c.** Tornado plot of model parameters varied in univariate sensitivity analysis of Index-Linked Testing via Home-Based modality and impact on Cost per Test. **d.** Tornado plot of model parameters varied in univariate sensitivity analysis of Index-Linked Testing via Caregiver modality and impact on Cost per Test

All 4 HIV testing unit costs (SoC and ILHIVT via 3 modalities) were most sensitive to changes in the conversion rate. Figures 2a *–* d present the results of the sensitivity analysis performed on the remaining variables. With regards to resource use inputs, personnel salaries had the largest influence on SoC unit costs (±3–7%), followed by overheads and the addition of up to 4 refresher trainings. Changes to capital resource inputs had a negligible effect on SoC unit costs. When considering the resource use inputs for ILHIVT across all three modalities, the unit cost per test was most sensitive to changes in clinic attendee HIV prevalence (±9–13%), followed by personnel salaries for all 3 modalities .

The impact of changing the parameters on cost per diagnosis unit costs are presented in Supplementary Fig. 1. Cost per diagnoses were most sensitive to changes in the conversion rate. Of the resource use inputs, personnel costs had the largest influence on SoC unit costs, while changes to clinic attendee HIV prevalence as well as testing yield, had the largest influence on ILHIVT related unit costs.

### Scenario analysis

The results of varying modality uptake and the subsequent impact on unit cost per test, compared to observed unit costs for the 4-month period in this study are presented in Table 4. If all 3 modalities have equal uptake, the unit cost of home-based testing increases and caregiver-provided testing becomes cheaper than clinic testing at urban clinics. If clinic and caregiver-provided testing have equal uptake, the unit cost of clinic testing decreases at both urban clinics but increases at the rural clinic, while unit costs of caregiver-provided testing at all three clinics decrease substantially. If all index-linked testing is via caregiver-provided testing, the cost per test at Clinic A, B and C respectively, are as follows: US$2.12, US$6.52, US$5.32.

**Table 4:** Scenario analysis of varying index-linked modality preference/uptake across all clinics: change in Unit Cost – Cost per Test

	Clinic	Home-Based	Caregiver
**Clinic A – Original Unit Cost**	**7.41**	**6.69**	**17.79**
Equal − 1/3 Distribution	$9.10	$8.85	$6.36
50:50 –Clinic/Caregiver	$6.37	N/A	$4.24
Caregiver Only	N/A	N/A	$2.12
**Clinic B – Original Unit Cost**	**31.08**	**7.18**	**21.63**
Equal −1/3 Distribution	$15.89	$13.32	$12.97
50:50 –Clinic/Caregiver	$10.89	N/A	$9.75
Caregiver Only	N/A	N/A	$6.52
**Clinic C – Original Unit Cost**	**5.36**	**8.65**	**62.40**
Equal −1/3 Distribution	$8.42	$10.29	$9.36
50:50 –Clinic/Caregiver	$5.92	N/A	$7.34
Caregiver Only	N/A	N/A	$5.32

In order for the caregiver – provided testing incremental unit cost per test to equate to that of ILHIVT via the home-based testing modality observed in the same 4-month time period, 163, 241 and 177 caregiver-provided tests would need to be administered at clinics A, B and C respectively. If all participants at clinics A and C who chose home-based testing had opted for caregiver-provided testing instead, it might have been possible to observe the lower situational caregiver – provided testing unit cost per test.

## Discussion

Our findings show that 6 times more children/adolescents were tested via ILHIVT compared to SoC HIV testing over the observed period. HIV prevalence among children/adolescents accessing SoC HIV testing (4%), was much higher than children/adolescents (0.7%) tested via ILHIVT. The cost of SoC HIV testing was lower in urban than rural settings, due to a greater number of tests administered at the urban clinics (44–71% more) and larger catchment populations. The cost of ILHIVT modalities were dependent on, and varied according to uptake. Costs involved in delivering SoC HTS were primarily driven by personnel followed by consumables. Whereas personnel followed by transaction costs were the largest drivers of ILHIVT costs at both urban and rural clinics. While management costs will be an important component of ensuring quality as well as accountability in strategies such as this that rely on identifying potential cases and outreach to the community, the management of this type of service delivery may need to be streamlined in an effort to keep transaction costs low.

The cost of delivering HIV testing through standard clinic-based services in this study ranged from US$5.90 to US$7.15, and is comparable to recent STAR project estimates from Malawi, Zambia and Zimbabwe (2016 US$4.24 to US$7.65) [22], yet higher compared to the results of a systematic review evaluating the cost of HTC in South Africa (2017/2018 US$3.62) [23]. The cost per positive diagnosis through standard clinic services in this study was lower than STAR estimates (US$73.63 to US$178.92) and likely reflects the higher HIV prevalence of those accessing testing among our study population (13%), compared to the STAR project (7%) [22].

In contrast, the high cost per diagnosis associated with ILHIVT was due to a low prevalence of HIV among index-linked children. An estimated > 90% of children living with HIV are vertically infected; those who were born prior to the scale up of prevention of mother to child transmission (PMTCT) programs are now likely to be adolescents and would therefore be diagnosed only when symptomatic, resulting in worse outcomes than those diagnosed and initiated on treatment in infancy [13, 24]. SoC HIV testing is likely to diagnose children with advanced disease, whereas asymptomatic children may not be brought to the clinic. Other studies of the same age-group in Zimbabwe found higher prevalence (2.6–15%) [11, 25], and index-linked testing of sexual partners and biological children in 3 rural provinces found a 30% HIV prevalence [12]. A study in Cameroon where only 46.2% of indexes consented to have their children tested, diagnosed HIV infection in 6.8% of children tested [24], while another index-linked study in Lesotho found an HIV prevalence of 1.8% among biological children of indexes [26]. The low HIV prevalence among index cases at the B-GAP clinics is likely attributable to recent HIV testing campaigns potentially resulting in saturation of testing, including a large Population Services International testing campaign operating in the area and exclusion of children/adolescents tested > 6 months prior to B-Gap screening.

HIV case finding will become more costly as knowledge of HIV status and treatment coverage increase. Alternative community based HIV testing strategies such as home-based testing and HIV Self-Test have been evaluated using costing studies [19, 27, 28], but this is the first costing study to evaluate index-linked testing, a strategy proposed by the WHO in order to expand HTC [29]. Our results provide the first unit costs of the different ILHVIT modalities for sub-Saharan Africa but they do not provide information on the cost-effectiveness of the strategy. While a full economic evaluation is necessary to accurately estimate the cost-effectiveness of ILHIVT, Phillips et al. (2019) have shown that cost per diagnosis can be used as a proxy for cost-effectiveness of HIV testing programs, and estimated a value for the cost-effectiveness threshold (2018) US$315 per HIV diagnosis [30]. By this definition, none of the index-linked modalities in our analysis would be considered cost-effective as the lowest cost per diagnosis estimated was US$385.35. However, our sensitivity analysis illustrated that when ILHIVT yield was increased from 0.7 to 7%, (as observed in Cameroon [24]), all but two modalities (clinic testing at an urban clinic, and rural caregiver-provided testing), resulted in cost per diagnoses below that US$315 threshold. ILHIVT cost per diagnosis would be more cost-effective if targeted to a higher-prevalence population. For example, a more efficient solution for scaling up ILHIVT could be to test the entire household; costs would be shared across a greater number of tests and yield would likely increase as adults have a higher prevalence of HIV compared to children/adolescents – although measures would need to be in place to ensure services screen out those previously diagnosed and on ART [27], and those testing are effectively linked to HIV treatment or prevention. A scale-up of any version of ILHIVT – in its current format, or expanded to the entire household – to either the regional or national level, requires careful consideration of the implications of potential (dis) economies of scale as well as other cost drivers. While this study explored drivers of cost at the facility level, and expected uptake is critical to this setting, physical capacity and infrastructure investment costs necessitate scrutiny at the regional or national level [31]. Furthermore, costs involved in geographical variability with regards to transport, training, management, monitoring and evaluation, quality assurance, as well as socio-cultural variability and acceptability affecting demand, must be considered [32].

HIV self-testing has the advantages of convenience, discretion and confidentiality, compared to clinic based HTS [33, 34]. HIV self-test has shown high acceptability and uptake elsewhere which resulted in WHO guidelines recommending scale-up [19–21]. In this study ‘self-test’ however referred to caregiver assisted testing of a child/adolescent. Caregiver uncertainty about correctly administering the test to a child without the aid of a health care professional, as well as fear of how to counsel a child with a positive result, could be factors accounting for the low levels of uptake within this study [35]. SoC clinic based HIV testing, when supplemented with HIV self-testing, can be a cost-effective testing option in a population with a high HIV prevalence and has the capacity to extend coverage rates, and therefore diagnoses [28, 33]. In this study, because assisted-testing had very low uptake, the incremental unit costs were 3.0 times greater than the cheapest modality (home–based) in an urban setting, and 11.6 times higher than the cheapest modality (clinic) in a rural setting. This low uptake raises questions around the differences of administering a self-test to someone else, compared to self-administration. Exploration into the dynamics and differences between HIV self-test and assisted self-test are needed.

To our knowledge, this study is the first to estimate the cost of ILHIVT in any setting. A limitation is that only 3 clinics were included. Although both urban and rural clinics are represented, showing that the cost and efficiency of each modality varies according to setting, it should be noted that the urban and rural clinics were located in different districts. While Bulawayo is an entirely urban district, and Mangwe an entirely rural district, there may be unique structural and/or district level factors which also influence unit costs. Additionally, the study was conducted within an economically unstable context. Zimbabwe dollarized its economy in 2008 following hyperinflation. In 2018 it introduced its own currency (RTGS), which triggered rapid inflation. In this study all RTGS amounts were converted to USD at the prevailing rate. Costs were highly sensitive to conversion rate as a result of the rapid inflation of RTGS against the US dollar.

## Conclusion

Innovative and alternative HIV testing strategies above SoC approaches are necessary in order to reach children and adolescents, given the additional barriers they face in order to get tested. The results of our study confirm that both costs and uptake of ILHIVT vary with setting. In addition, while ILHIVT allows for greater access of this difficult to reach population, compared to the standard method, uptake is a key driver of the cost per test. To ensure efficiency when scaling up ILHIVT, acceptability of testing modality needs to be considered and alternative index linked testing solutions that increase yield such as ILHIVT of the entire household, as opposed to solely targeting children/adolescents, need to be explored. Additionally, there is potential to benefit from economies of scope by integrating ILHIVT with other activities, however this would require further consideration and research.

### Supplementary Information

**Additional file 1: Supplementary Figure 1**. Sensitivity Analysis: Tornado Plots Illustrating Parameter Impact on Cost per Diagnosis.

## Data Availability

The datasets during and/or analyzed during the current study available from the corresponding author on reasonable request.

## Appendix 1

### Summary of time tracking diaries for ILHIVT activities: compiled via direct observation and self-evaluation

Time tracking diaries completed by the costing team through direct observation, as well as time tracking diaries completed by research assistants through self-evaluation had the same format: an activity code, a description of the activities and a table which divided the workday into half hour increments. The following illustrates an abbreviated version of how human resource use was delineated and recorded.

**Instructions:** Using the activity codes below, please record the appropriate activity for each half hour interval of your work day, over the course of one consecutive week. If activities conducted do not fall into one of the pre-listed activities below, please specify the activity in the blank codes (code 13–15), and enter into the table.

**Table:**

Activity Code	Description of Activity
**01**	***Organization/Administrative Activities*** (Ex. Preparing CRFs for use the next day, photo copying, filing, filling out petty cash ledger etc.)
**02**	***Screening Index Cases***, Including morning health talk and evaluating self-testing competency
**03**	***Testing Children/Adolescents*** of Index Cases
**04**	***Filling out CRFs*** (All CRFs: Enrolment, Outcome, Locator, etc.)
**05**	***Follow-Up***, including phone calls made for testing reminders, initiating testing & following-up of results with community partners, linking to treatment, enrolling in CHW intervention, etc.
**06**	***Outcome Assess*****ment**, including exit interview, sample collection, sending sample out for processing
**07**	***Meetings*** (Ex. With study coordinator; at clinic; with community partners; with stakeholders)
**08**	Assisting clinic staff with ***non-B-Gap related*** tasks (Ex. Testing O/I patients)
**09**	***Breaks*** and ***Down-Time*** (Ex. During a slow day, waiting for patients etc.)
**10**	***Community Testing*** (Home Visits)
**11**	***Travelling*** to households for community testing
**12**	***Collection*** of ***Assisted Self-Test*** kits
**13**

**Table:**

Time of Day	Monday	Tuesday	Wednesday	Thursday	Friday
08:00–08:30					
08:30–09:00					
Etc.					

A total of 56 direct observation hours and 535 self-evaluation hours were recorded via the time tracking diaries. Daily activity breakdown is classified into three categories and presented below: Clinic Hours; Community Hours; Administration

**Table:**

Allocation of Human Resource Use Involved in ILHIVT Activities: Percent of Time Spent			
Clinic	Clinic Hours	Community Hours	Administration
A – Bulawayo (Urban)	20.3%	20.6%	59.1%
B – Bulawayo (Urban)	22.1%	11.3%	66.7%
C – Mangwe (Rural)	7.3%	22.8%	70.0%

## Appendix 2

**Table 5:** Detailed Description of Cost Resource Collection: Methods

Cost Category	Primary Method of Data Collection	Details
*Recurrent Costs*		
Personnel	**HTS:** Interviewing the Nurse-In-Charge **Index-Linked-Testing:** Interviews with the study coordinator and research assistants, in addition to direct observation	**HTS:** - When all clinic staff was recorded, clinic staff time was measured through direct observation, with a particular focus on the HTS. - A PCC at each clinic was observed for a full work day, by two separate individuals, and was also interviewed. **Index-Linked-Testing:** - Study personnel demarcate their time solely to index-linked testing activities. As a result time-tracking diaries were completed by study personnel to detail time spent on specific activities over the course of the study, and directly observed while in clinic also. - 6 research assistants completed diaries spanning 2 weeks each. - Direct observation was completed by 2 separate individuals, observing each of the 6 assistants for 1 day each.
Consumables	**HTS:** Interviewing the primary care counsellors **Index-Linked-Testing:** Interviewing study research assistants	**HTS:** First, PCCs were asked to specify every consumable which was required for an HTS client. Then each item was catalogued by natural unit – ex. 500 g bag of absorbent cotton, 1 roll per bag. Following this, each consumable was quantified per consumption length – ex. one pack of cotton lasts on average, 3.5 weeks. Use was then calculated monthly (ex. 1.14 pack/month), and monthly use was then extrapolated to cover a one-year time period. **Index-Linked-Testing:** Method, same as above.
Training	**HTS:** Interviewing the Nurse-in-Charge/District Medical Officer **Index-Linked-Testing:** Abstracting study accounting files and interview with study coordinator.	Frequency of training and refresher training sessions was noted.
Transportation/ Duties	**Index-Linked-Testing** (only)**:** Abstracting study accounting files	Frequency and amount of duties related to index-linked testing consumables was noted.
Overheads	**HTS:** Interviewing nurse-in-charge **Index-Linked-Testing:** Abstracting study accounting files	**HTS:** Overheads were collected and then apportioned according to HTS usage, compared to the size of the other clinic departments. **Index-Linked-Testing:** Abstracting study accounting files.
Lab-Processing	**Index-Linked-Testing** (only)**:** Abstracting study accounting files	Frequency and amount of processing fees related to viral load suppression assessment was noted.
*Capital Costs*		
Building/ Facility Space	**HTS:** Direct observation and physically pacing/spacing the clinic.	The area of the clinic and HTS were manually measured.
Furniture and Equipment	**HTS:** Direct observation and recording **Index-Linked-Testing:** Direct observation, recording and abstracting study accounting files	Furniture and equipment directly involved in service provision were physically counted and recorded.
Intervention Start – Up	**Index-Linked-Testing** (only)**:** Abstracting study accounting files	Frequency and financial resources invested in initial RA training, as well as RA rapid diagnostic and OMT testing training, was noted.

## Appendix 3

**Table 6:** Detailed Description of Cost Resource Collection: Interviews

Interviewee	Purpose
*HTS*	
1 Nurse in Charge at each of the 3 clinics (supplemented by at least one other RGN at each clinic)	To obtain: - Rundown of all clinic personnel and their roles - All inputs included in clinic overheads
1 Primary Care Counsellor at each of the three clinics	To obtain: - Activity breakdown of HTS - Resource data collection involved in HTS
Assistant Director of Bulawayo City Council (BCC) and BCC finance department	To obtain: - Bulawayo clinic personnel salary schedule - Costs per training/refresher training workshops related to HTS - All overhead costs
Provincial Medical Director and MAT South finance department	To obtain Mangwe clinic personnel salary schedule
District Medical Director (Mangwe)	To obtain: - Salary for district’s HIV focal person - Costs per training/refresher training workshops related to HTS
District Environmental Health Officer (Mangwe)	To obtain Mangwe clinic overhead costs
MAT South Pharmacist	To obtain HTS consumable costs
*Index-Linked Testing*	
Research Coordinator	To understand overall flow of study, obtain outcome data, query clarifications
6 Research Assistants; 2 at each of the three clinics	To discern daily activity breakdown and recurrent resources consumed over the course of study activities
BRTI Study Accountant	To obtain all study related costs: - RA salary schedule - Study consumable costs - Recurrent costs: Overheads and Training
BRTI Administrator	To obtain equipment costs

## Abbreviations

HIV: Human Immunodeficiency Virus
ART: Antiretroviral Therapy
ILHIVT: Index-Linked HIV Testing
WHO: World Health Organization
B-GAP: Bridging the GAP in HIV Testing and Care for Children in Zimbabwe
SoC: Standard of Care
HTC: HIV Testing and Counselling
OMT: Oral Mucosal Transudate
RTGS: Real-Time Gross Settlement
USD: United States Dollars
GDP: Gross Domestic Product
NGO: Non-Governmental Organization
