# Supplementary material for: A costing analysis of B-GAP: index-linked HIV testing for children and adolescents in Zimbabwe
Source: BMC Health Serv Res. 2021 Oct 12;21:1082. doi: 10.1186/s12913-021-07070-3 (PMC8507161; doi:10.1186/s12913-021-07070-3)
Supplement: Supplementary file 1 — Additional file 1: Supplementary Figure 1. Sensitivity Analysis: Tornado Plots Illustrating Parameter Impact on Cost per Diagnosis. [file 12913_2021_7070_MOESM1_ESM.docx]

Supplementary Figure 1 – Sensitivity Analysis: Tornado Plots Illustrating Parameter Impact on Cost per Diagnosis

**Figure S1b**. Tornado plot of model parameters varied in univariate sensitivity analysis of Index-Linked Testing via **Clinic** modality and impact on **Cost per Diagnosis** (Note: Conversion rate variable omitted)

**Figure S1d**. Tornado plot of model parameters varied in univariate sensitivity analysis of Index-Linked Testing via **Caregiver** modality and impact on **Cost per Diagnosis** (Note: Conversion rate variable omitted)

**Figure S1c**. Tornado plot of model parameters varied in univariate sensitivity analysis of Index-Linked Testing via **Home-Based** modality and impact on **Cost per Diagnosis** (Note: Conversion rate variable omitted)

**Figure S1a**. Tornado plot of model parameters varied in univariate sensitivity analysis of Adolescent **SoC HTS** and impact on **Cost per Diagnosis.**

(Note: Conversion rate variable omitted)
